# The human impact of commercial delivery cycling injuries: a pilot retrospective cohort study

**DOI:** 10.1186/s40814-022-01077-1

**Published:** 2022-06-01

**Authors:** Mitchell N. Sarkies, Cameron Hemmert, Yu-Chen Pang, Christine T. Shiner, Karon McDonell, Rebecca Mitchell, Reidar P. Lystad, Michael Novy, Lauren J. Christie

**Affiliations:** 1 Centre for Healthcare Resilience and Implementation Science, Australian Institute of Health Innovation, Faculty of Medicine, Health and Human Sciences, Macquarie University, 75 Talavera Road, Sydney, NSW 2109 Australia; 2 Department of Health Professions, Faculty of Medicine, Health and Human Sciences, Macquarie University, Sydney, Australia; 3 Departments of Rehabilitation and Pain Medicine, St Vincent’s Hospital Sydney, Sydney, Australia; 4 St Vincent’s Clinical School, Faculty of Medicine, University of New South Wales, Sydney, Australia; 5 Trauma Service, St Vincent’s Hospital Sydney, Sydney, Australia; 6 Allied Health Research Unit, St Vincent’s Health Network, Sydney, Australia; 7 Nursing Research Institute, St Vincent’s Health Network, Sydney, Australia

**Keywords:** Accident prevention, Accidents, Traffic, Injuries, Occupational health, Road, Safety management, Cycling, Commercial, Delivery

## Abstract

**Background:**

Commercial delivery cyclists represent a uniquely vulnerable and poorly understood road user. The primary aim of this study was to pilot whether cycling injuries could be categorised as either commercial or non-commercial using documentation entered into routine hospital medical records, in order to determine the feasibility of conducting a large cohort study of commercial cycling injuries in the future. A secondary aim was to determine which key demographic, incident and injury characteristics were associated with commercial versus non-commercial cycling injuries in emergency.

**Methods:**

Pilot retrospective cohort study of adults presenting to an acute public hospital emergency department between May 2019 and April 2020 after sustaining a cycling-related injury. Multinomial logistic regression was used to examine the demographic, incident and injury characteristics associated with commercial compared to non-commercial cycling.

**Results:**

Of the 368 people presenting to the emergency department with a cycling-related injury, we were able to categorise 43 (11.7%) as commercial delivery cyclists, 153 (41.6%) as non-commercial cyclists and the working status of 172 (46.7%) was unable to be confirmed. Both commercial and unconfirmed cyclists were more likely to be younger than non-commercial cyclists. Compared to non-commercial cyclists, commercial cyclists were 11 times more likely to speak a language other than English (AOR 11.3; 95% CI 4.07–31.30; *p*<0.001), less likely to be injured from non-collision incidents than vehicle collisions (AOR 0.36; 95% CI 0.15–0.91; *p*=0.030) and were over 13 times more likely to present to the emergency department between 8.00pm and 12.00am compared to the early morning hours (12.00 to 8.00am) (AOR 13.43; 95% CI 2.20–82.10; *p*=0.005).

**Conclusions:**

The growth of commercial cycling, particularly through online food delivery services, has raised concern regarding commercial cyclist safety. Improvements in the recording of cycling injury commercial status is required to enable ongoing surveillance of commercial cyclist injuries and establish the extent and risk factors associated with commercial cycling.

## Key messages regarding feasibility

- What uncertainties existed regarding the feasibility?
  It was uncertain whether medical record review could be used to supplement other routinely collected hospital administrative data to categorise cycling injury emergency department presentations as either commercial or non-commercial cycling.
- What are the key feasibility findings?
  Just over half of the cycling injuries were able to be categorised as commercial or non-commercial using medical record review and other routine hospital administrative data sources.
- What are the implications of the feasibility findings for the design of the main study?
  A substantial proportion of cycling injury emergency department presentations are unable to be categorised as commercial or non-commercial using existing data sources. Targeted data collection in the form of a prospective observational study and ongoing injury surveillance is required to establish and monitor the extent and risk factors associated with commercial cycling.

## Background

Internationally, vulnerable road users have not experienced the same improvements in safety achieved for motor vehicle road users, particularly in car-dependent countries such as the US and Australia [1, 2]. Each year in Australia, an average of 38 people are killed and 12,000 injured in transport-related incidents while cycling [3]. Concerningly, the rate of cycling-related hospitalisations for major trauma has increased by approximately 8% each year between 2007 and 2015 [2], while the rate of injury-related hospitalisations of other road users has reduced over the same period [2, 3]. These findings are consistent with trends observed in similar countries, like the US which experienced an 11% rise in the per-capita cyclist fatality rate between 2010 and 2018 [1].

The rapid growth in the online food delivery industry, particularly in major cities, has driven a rise in commercial delivery cyclists who represent a uniquely vulnerable population of road users [4]. Concerns have been expressed regarding poor working conditions, reports of coercion and exploitation, limited training and risks to safety experienced by commercial delivery cyclists [5]. Currently, there is limited understanding of the characteristics, behaviours, injuries and health impact of commercial delivery cycling [6–8]. It is possible that commercial delivery cycling may introduce additional risks of injury compared to non-commercial cycling, which may influence injury patterns. Commercial delivery cyclists are typically incentivised to perform deliveries quickly [9], their delivery distance range can extend up to 10km [10] and the combination of these two factors can constrain the ability of commercial cyclists to make proactive route choices that would avoid high volume traffic, potentially increasing the risk of motor vehicle collisions compared with people cycling recreationally. One study from New York City reported that nearly 35% of all injuries sustained while riding a bicycle occurred while working despite there being a mandatory road safety training program for commercial cyclists [11]. These commercial cyclists were predominantly young males from minority ethnic backgrounds who were less likely to wear helmets or be distracted by electronic devices [11].

Commercial delivery cyclists must contend with an underlying conflict between safety and working conditions. In China, risky road behaviours and traffic violations in commercial delivery cyclists have been associated with higher injury severity [12]. Explanations for these risky road behaviours are nuanced and depend on the individual profiles of the cyclists. For example, Papakostopoulos and Nathanael report that those trying to cope with work pressures may run red lights, whereas those trying to maximise profit are associated with helmet non-use [13]. Red light running is thought to be influenced by high intensity of work over long hours, lack of breaks and high levels of stress experienced by commercial delivery cyclists [14–16]. These issues would require a different set of solutions to say helmet non-use, which may be driven by time saving and convenience [16]. However, these findings are likely to be specific to local context and infrastructure, which limits their generalisability and potential relevance to other settings such as Australia.

In New South Wales, Australia, road crash data can be used to identify whether injured cyclists were riding commercially or non-commercially. However, this data is not a representative sample of the cohort of injured cyclists because it only covers incidents that are reported to the police, occur on public roads, involve at least one moving road vehicle, and one person being injured or killed or at least one motor vehicle being towed away. Data could also be collected from hospital emergency departments to ensure road incidents and cycling injuries that do not meet the road crash data criteria are observed. It is unclear whether routine hospital administrative data collections and patient medical records contain sufficient information to enable cycling injuries to be categorised as either commercial or non-commercial. The trauma impact of commercial cycling injuries requires investigation for this vulnerable population, as even minor injuries can result in short-term productivity losses and increased health service utilisation. The primary aim of this study was to pilot whether cycling injuries could be categorised as either commercial or non-commercial using documentation entered into routine hospital medical records, in order to determine the feasibility of conducting a large cohort study of commercial cycling injuries in the future. A secondary aim was to determine which key demographic, incident and injury characteristics were associated with commercial versus non-commercial cycling injuries in emergency. These aims were to be addressed by achieving the following objectives:

Identify the proportion of cycling injuries that could be categorised as either commercial, non-commercial or unconfirmed.

Examine the relationship between demographic, incident and injury characteristics with commercial versus non-commercial cycling injuries.

## Methods

### Design and setting

A pilot retrospective cohort study was conducted at St Vincent’s Hospital, Sydney, Australia, over a 12-month period (May 2019 to April 2020). The study site was a 400 bed, acute public hospital, which provides a trauma care service for metropolitan Sydney, including the central business district. The study was approved by St Vincent’s Hospital Human Research Ethics Committee (REF 2020/ETH02642).

### Study population and sample

All adults aged ≥18 years who presented to the hospital emergency department (ED) within the 12-month period were included in the study. Eligible cases were identified from the local trauma registry and ED information system using the search terms ‘bike’, ‘cycle’, ‘push-bike’ and ‘cyclist’. Two investigators (CH and YCP) manually screened records for eligibility and extracted the data into the Research Electronic Data Capture (REDCap) tool for secure storage. Records were excluded if the primary reason for the ED presentation was not cycling-related, for example motorcycle-related transport incidents. Commercial cycling status was unable to be easily distinguished using routinely collected administrative data as these fields often contained incorrect or missing information. Therefore, a manual review of medical records was conducted, supplemented by documentation in the hospital’s patient management system to distinguish between commercial and non-commercial cycling status.

#### Commercial cycling status

Cycling-related injuries were categorised as either commercial, non-commercial or unconfirmed working status. Confirmation of commercial cycling was determined if the incident was explicitly documented to have occurred while working, for example ‘riding for Uber Eats’. Administrative data that indicated the activity at time of injury and financial classification (e.g. workers’ compensation) were also used to distinguish between commercial and non-commercial cyclists in some instances.

Cycling was deemed ‘non-commercial’ if the reason for cycling was explicitly outlined as recreational or travel that was not undertaken for occupation, including where the patient’s occupation was documented as not related to cycling (e.g. ‘patient employed as an accountant’). Records were assigned as unconfirmed working status when there was no explicit documentation on the reason for cycling, for example ‘fell off bike’.

#### Demographic, incident and injury characteristics

Demographic characteristics included the age, sex, primary language and financial classification (i.e. how the hospital episode was funded). The incident characteristics were place of injury, external cause of injury, motor vehicle type (where applicable), bike type, helmet use, ambulance scene attendance and ED arrival month and time of day. Injury characteristics included the ED triage category, number of injuries, nature of injury, the Abbreviated Injury Scale (AIS) [17], injured body region count and Injury Severity Score (ISS) [18], whether trauma team activation was required, operative procedure requirement, initial total Glasgow Coma Scale (GCS) [19] and post-ED disposition.

### Management of potential risk of bias

Instances of uncertainty when classifying commercial status were discussed between two investigators (CH and YCP) to reach a consensus. When consensus was not met, a third investigator (LC) was consulted and a final decision on classification was made. A conservative approach was taken towards classification whereby cases were recorded as ‘unknown working status’ if there was any uncertainty.

### Data analysis

All analyses were conducted using SAS version 9.4 (SAS Institute, Inc.; Cary, NC). Descriptive statistics were presented as a mean and standard deviation or number and percentage. Multinomial logistic regression was performed to examine the relationship between the demographic, incident and injury characteristics (independent variables) for commercial, non-commercial and cyclists with an unconfirmed working status (dependent variable) presenting to the ED. Variables were included if they had been previously associated with injuries sustained while commercial delivery cycling [11], were collected in the local trauma registry and were statistically significant during univariate analysis (i.e. age, primary language, injury cause, and time of ED arrival). A forward stepwise regression was used to select variables that significantly contributed to the model. Two-way interactions were also examined. A statistical significance was set at a p-value ≤0.05. The effect size was presented as an adjusted odds ratio (AOR) with 95% confidence intervals (CI).

## Results

Of the 368 cycling-related ED presentations, we were able to categorise 43 (11.7%) as commercial delivery cyclists, 153 (41.6%) as non-commercial cyclists and the working status of 172 (46.7%) was unable to be confirmed. Most people injured were male regardless of their working status. The commercial cyclists had a lower mean age and fewer spoke English as their primary language, compared to both the non-commercial cyclists and cyclists with an unconfirmed working status. ED presentations were predominantly government funded through Medicare for both the non-commercial cyclists and cyclists with an unconfirmed working status; however, the commercial cyclists’ were more often funded by the compulsory third-party insurance scheme (Table 1).

**Table 1:** Demographic characteristics of injured cyclists presenting to a hospital ED by working status, May 2019 to April 2020

Characteristic	Commercial cycling (*n*=43)	Non-commercial cycling (*n*=153)	Unconfirmed commercial cycling (*n*=172)
Age, *mean (SD)*	26.14 (7.9)	46.23 (14.5)	38.00 (13.5)
Sex, *n (%)*			
Male	31 (72.1)	119 (77.8)	135 (78.5)
Female	12 (27.9)	33 (21.6)	35 (20.4)
Indeterminate/intersex/unspecified	0 (0.0)	1 (0.7)	2 (1.2)
Primary language, *n (%)*			
English	11 (25.6)	140 (91.5)	127 (73.8)
Spanish	10 (23.3)	8 (5.2)	23 (13.4)
Other	21 (48.8)	5 (3.3	22 (1.2)
Financial class, *n (%)*			
Public, Medicare	3 (6.9)	99 (64.7)	83 (48.3)
Private	0 (0.0)	15 (9.8)	7 (4.1)
Workers’ compensation	8 (18.6)	0 (0.0)	0 (0.0)
Compulsory third party insurance	16 (37.2)	29 (18.9)	50 (29.1)
Overseas visitor	5 (11.6)	6 (3.9)	5 (2.9)
Medicare ineligible patient	11 (25.6)	4 (2.6)	27 (15.7)

**Fig. 1:**
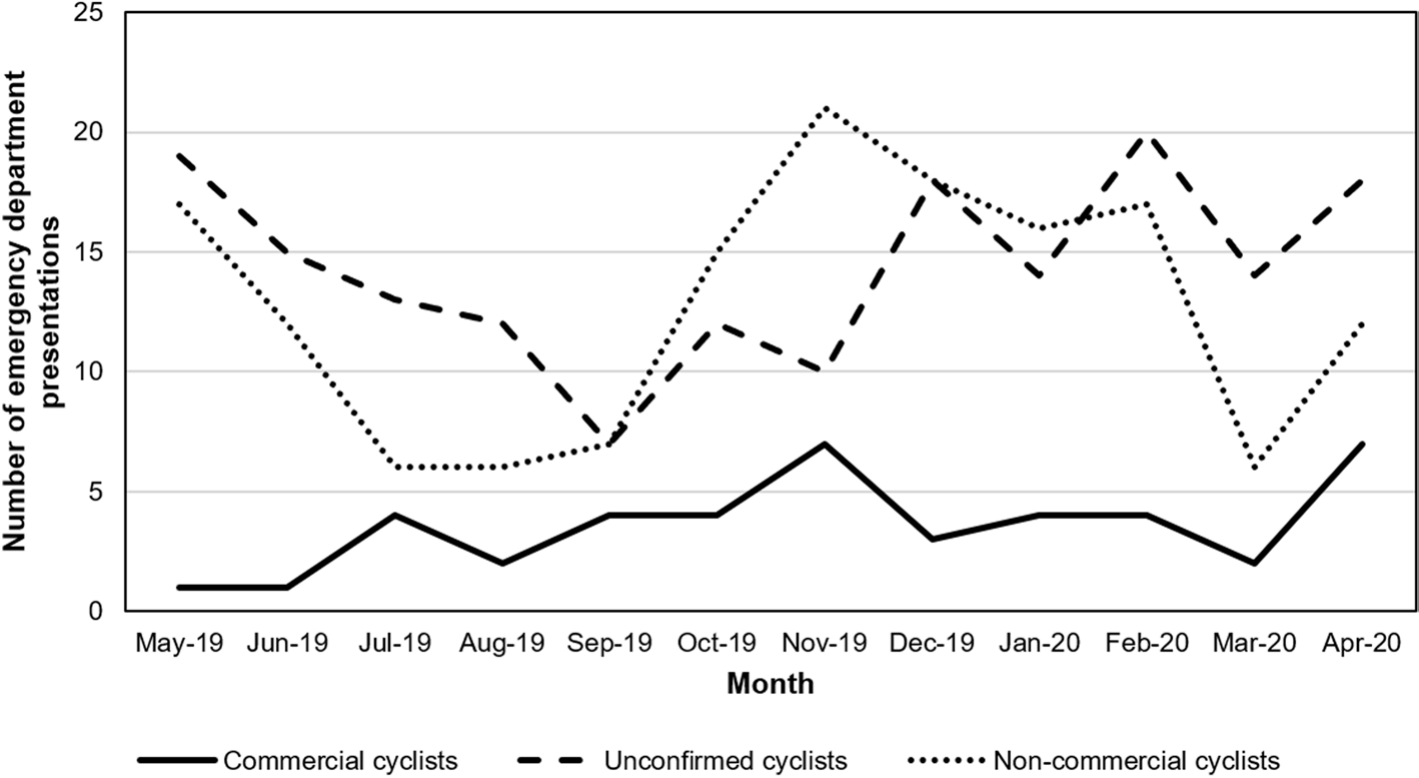
Number of injured cyclists presenting to the emergency department by month and working status, May 2019 to April 2020. *X*-axis refers to the number of emergency department presentations. *Y*-axis refers to the month. The solid line refers to the commercial cyclists. The large dash line refers to unconfirmed cyclists. The small dash line refers to non-commercial cyclists

**Fig. 2:**
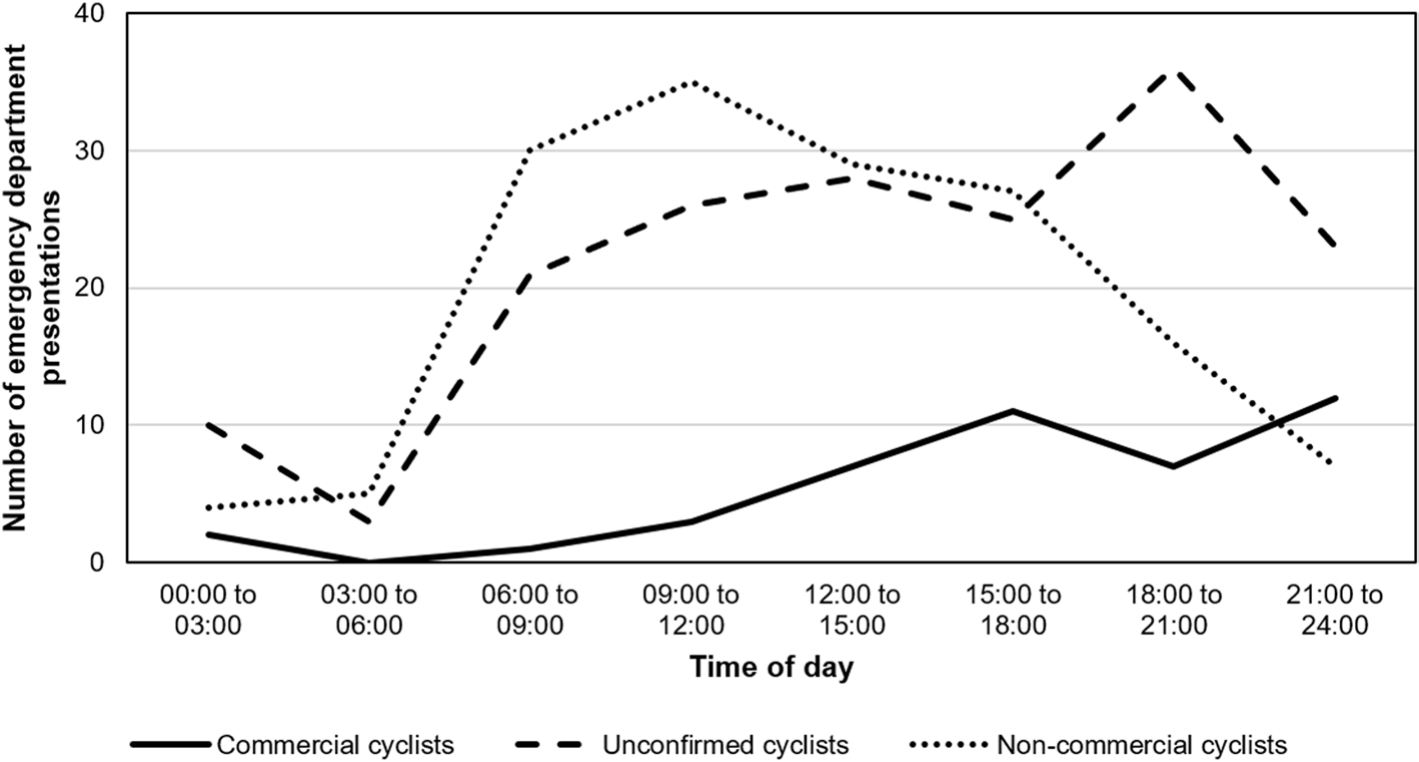
Number of injured cyclists presenting to the emergency department by time of day and working status, May 2019 to April. *X*-axis refers to the number of emergency department presentations. *Y*-axis refers to the time of day. The solid line refers to the commercial cyclists. The large dash line refers to unconfirmed cyclists. The small dash line refers to non-commercial cyclists

Non-collision-related cycling incidents (e.g., falling while getting on or off bicycle) were among the most frequent injury mechanism for all three cycling categories, although non-collisions were more common for non-commercial (54.3%) compared to commercial (34.9%) cyclists. There was a larger proportion of commercial cyclists who were injured while riding on a roadway and struck by a motor vehicle than non-commercial cyclists or those with an unconfirmed working status. Commercial cyclists were also more often wearing a helmet and using an E-bike than both non-commercial cyclists and those with an unconfirmed working status (Table 2). The number of ED presentations across each month of the year and time of day are presented in Figs. 1 and 2, respectively.

**Table 2:** Incident characteristics of injured cyclists presenting to a hospital ED by working status, May 2019 to April 2020

Characteristic	Commercial cycling (*n*=43)	Non-commercial cycling (*n*=153)	Unconfirmed commercial cycling (*n*=172)
Repeat ED presentations, *n (%)*	8 (18.6)	18 (11.8)	18 (10.5)
Ambulance scene attendance, *n (%)*	15 (34.9%)	60 (39.2%)	53 (30.81%)
ED triage category, *n (%)*			
Category 1^a^	3 (6.9)	13 (8.5)	18 (10.5)
Category 2^b^	11 (25.6)	35 (22.9)	39 (22.47)
Category 3^c^	10 (23.3)	53 (34.6)	47 (27.3)
Category 4^d^	17 (39.5)	46 (30.1)	59 (34.3)
Category 5^e^	0 (0.0)	1 (0.7)	2 (1.2)
Not specified	2 (4.7)	5 (3.3)	7 (4.1)
Place of injury, *n (%)*			
Driveway to home	0 (0.0)	3 (1.9)	1 (0.6)
Roadway	26 (60.5)	63 (41.2)	65 (37.8)
Sidewalk	1 (2.3)	6 (3.9)	7 (4.1)
Cycleway	1 (2.3)	4 (2.6)	0 (0.0)
Other roadway	6 (13.9)	27 (17.7)	37 (21.5)
Parking lot	0 (0.0)	1 (0.7)	0 (0.0)
Other	0 (0.0)	16 (10.5)	2 (1.2)
Not specified	8 (18.6)	33 (21.6)	61 (35.5)
Injury cause, *n (%)*			
Collision	24 (55.8)	51 (33.3)	73 (42.4)
Non-collision	15 (34.9)	83 (54.3)	69 (40.1)
Other and unspecified	4 (9.3)	19 (12.4)	30 (17.4)
Motor vehicle involved in incident, *n (%)*			
Car	13 (30.2)	36 (23.5)	52 (30.2)
Other (truck, bus, motorbike, scooter, taxi)	3 (6.9)	2 (1.3)	6 (3.5)
Not documented/not Specified	2 (4.7)	3 (1.9)	18 (10.5)
No vehicle	25 (58.1)	112 (73.2)	96 (55.8)
Helmet use, *n (%)*			
Yes	30 (69.8)	101 (66.0)	95 (55.2)
No	0 (0.0)	4 (2.6)	13 (7.6)
Unknown	13 (30.2)	48 (31.4)	64 (37.2)
Bike type, *n (%)*			
Push bike	33 (76.7)	140 (91.5)	144 (83.7)
E-bike	7 (16.3)	4 (2.6)	17 (9.9)
Unknown	3 (6.9)	7 (4.6)	11 (6.4)

^a^Injuries immediately life threatening

^b^Imminently life-threatening, important time critical condition

^c^Potentially life-threatening, situational urgency

^d^Potentially serious, situational urgency, complex presentation

^d^Less urgent, clinical administrative

All cycling categories had a higher proportion of non-orthopaedic injuries (e.g. lacerations, abrasions, soft tissue) and injuries to the extremities or pelvic girdle body regions. An ISS was available for one third of records; of those, non-commercial cyclists (9.27) had higher mean ISS and a smaller proportion of minor injuries (53.3%) compared with commercial cyclists (3.25 and 100%, respectively). Non-commercial cyclists received operative procedures more frequently than commercial cyclists. Commercial cyclists were more often discharged from the hospital ED with their treatment completed; although, they (18.6%) represented to the ED more often than both the non-commercial cyclists (11.8%) and those with an unconfirmed working status (10.5%) (Table 3).

**Table 3:** Injury characteristics of injured cyclists presenting to a hospital ED by working status, May 2019 and April 2020

Characteristic	Commercial cycling (*n*=43)	Non-commercial cycling (*n*=153)	Unconfirmed commercial cycling (*n*=172)
Number of injuries, *n*	59	225	246
Nature of injury, *n (%)*^i^			
Head^a^	10 (16.95)	33 (14.7)	37 (15.0)
Chest/abdominal^b^	0 (0.00)	18 (8.0)	14 (5.7)
Orthopaedic^c^	9 (15.25)	71 (31.6)	62 (25.2)
Other non-orthopaedic^d^	40 (67.80)	103 (45.8)	133 (54.1)
Number of body regions injured, *n*	63	248	267
Abbreviated Injury Scale body region, *n (%)*^j^			
Head or neck	10 (15.87)	37 (14.9)	42 (15.7)
Face	10 (15.87)	30 (12.1)	26 (9.7)
Chest	1 (1.59)	23 (9.3)	18 (6.7)
Abdominal or pelvic contents	0 (0.00)	4 (1.6)	7 (2.6)
Extremities or pelvic girdle	34 (53.97)	119 (47.9)	127 (47.6)
External^e^	10 (15.87)	35 (14.1)	45 (16.9)
Injury severity score available^f^, *n (%)*	12 (27.91)	45 (29.4)	63 (36.6)
Injury severity score, *mean (SD)*	3.25 (1.96)	9.27 (6.2)	5.40 (4.6)
Mild (<9), *n (%)*	12 (100)	24 (53.3)	48 (76.2)
Moderate (9–15), *n (%)*	0 (0.0)	14 (31.1)	11 (17.5)
Severe (16–25), *n (%)*	0 (0.0)	7 (15.6)	4 (6.3)
Profound (>25), *n (%)*	0 (0.0)	0 (0.0)	0 (0.0)
Tauma team consult^g^, *n (%)*	12 (27.91)	48 (31.4)	58 (33.7)
Operative procedure required^h^, *n (%)*	5 (11.63)	24 (15.7)	18 (10.5)
Glasgow Coma Scale, *mean (SD)*	14.97 (0.17)	14.87 (0.4)	14.95 (0.3)
Post-ED disposition, *n (%)*			
Discharged - treatment completed	34 (79.1)	99 (64.7)	112 (65.1)
Discharged - did not wait	1 (2.3)	5 (3.3)	9 (5.2)
Discharged - against advice	0 (0.0)	3 (1.9)	8 (4.7)
Admitted to acute hospital	7 (16.3)	39 (25.5)	35 (20.3)
Transferred to other facility	1 (2.3)	2 (1.3)	7 (4.1)

^a^Injuries involving cranial structures, such as post-trauma headaches, skull fractures and intracranial haemorrhage

^b^Injuries involving the chest, chest wall and abdominal structures, such as intraabdominal bleeds, rib fractures and pneumothorax

^c^Injuries such as fractures to extremities

^d^Injuries such as lacerations, abrasions, contusions and sprains

^e^AIS denotes classifying abrasions and lacerations as external. When the location of injury was explicitly stated we categorised injuries such as abrasions and lacerations under the body region stated in medical notes and categorised as external if not stated

^f^89 records excluded due to blank score

^g^Records ranged from consult with trauma staff to full trauma team activation

^h^Recorded if referred to operating suite for injuries sustained while cycling

^i^Refers to count of nature of injury categories

^j^Refers to count of injuries sustained in regions outlined abbreviated injury scale body regions

Both commercial cyclists and cyclists with an unconfirmed working status who were injured and presented to the hospital ED were significantly more likely to be younger than non-commercial cyclists. Compared to non-commercial cyclists, commercial cyclists were 11 times more likely to speak a language other than English (AOR 11.3; 95% CI 4.07–31.30; *p*<0.001) and cyclists with an unconfirmed working status were more than twice as likely to speak a language other than English (AOR 2.60; 95% CI 1.26–5.38; *p*=0.010). Commercial cyclists were less likely to be injured from non-collision incidents than collisions with vehicles, compared with non-commercial cyclists (AOR 0.36; 95% CI 0.15–0.91; *p*=0.030) as cyclists with an unconfirmed working status (AOR 0.51; 95% CI 0.30–0.86; *p*=0.011). Commercial cyclists were around 13 times more likely than non-commercial cyclists to present to ED between 8.00pm and 12.00am compared to the morning time period between 12.00 and 8.00am (AOR 13.43; 95% CI 2.20–82.10; *p*=0.005). Similarly, cyclists with an unconfirmed working status were more than 2.5 times more likely than non-commercial cyclists to present to ED between 8.00pm and 12.00am compared to the morning period between 12.00 and 8.00am (AOR 2.67; 95% CI 1.06–6.77; *p*=0.038) (Table 4).

**Table 4:** Multinomial logistic regression of demographic, incident, and injury characteristics by commercial cycling status

Characteristic	Commercial cycling (*n*=43)	Unconfirmed commercial cycling (*n*=172)
	Adjusted odds ratio & 95% CIs^a^	Adjusted odds ratio & 95% CIs^a^
**Age group**		
18–24	1	1
25–34	0.22 (0.07 to 0.71), 0.011	0.49 (0.19 to 1.28), 0.146
35–54	0.04 (0.01 to 0.19), 0.000	0.40 (0.16 to 1.03), 0.057
55+	0.03 (0.00 to 0.29), 0.002	0.19 (0.07 to 0.55), 0.002
**Primary language**		
English	1	1
Other	11.25 (4.06 to 31.19), 0.000	2.60 (1.26 to 5.38), 0.010
**Injury cause**		
Collision	1	1
Non-collision	0.36 (0.15 to 0.91), 0.030	0.51 (0.30 to 0.86), 0.011
Other and unspecified	0.74 (0.21 to 2.57), 0.637	1.00 (0.50 to 1.98), 0.999
**ED arrival time**		
Midnight-0759	1	1
0800–1459	4.45 (0.76 26.13), 0.098	1.30 (0.63 to 2.66), 0.479
1500–1959	4.51 (0.77 to 26.26), 0.094	1.78 (0.82 to 3.86), 0.143
2000–2359	13.43 (2.20 to 82.10), 0.005	2.67 (1.06 to 6.77), 0.038

^a^Non-commercial cycling was the referent group

## Discussion

This is the first pilot study exploring the impact of the food delivery gig-economy and commercial cycling in the Australian context. The study demonstrated that just over half of the cycling injuries presenting to the emergency department during the study period were able to be categorised as either commercial or non-commercial. Commercial cyclists share distinct demographic, incident and injury patterns that differ from non-commercial cyclists. Commercial and unconfirmed cyclists were more likely than non-commercial cyclists to be younger, speak a language other than English and present to ED after injury in the evening (8.00pm to 12.00am) compared to the morning (12.00 to 8.00am). Commercial cyclists and cyclists with an unconfirmed working status were both comparatively more likely to be injured from collision with a vehicle than non-commercial cyclists.

In addressing the primary aim of this pilot study, we highlight the need for improving data capture to better distinguish between commercial and non-commercial cyclists. Information obtained from the ‘activity when injured’ and ‘financial classification’ fields within the routinely collected hospital administrative registry data [20] was not adequate to identify commercial cyclists, requiring the research team to conduct a medical record review. Even by doing so, nearly half of cycling injury ED presentations were unable to be categorised as work-related or not. This constrains the ability of government regulatory agencies, employee representatives, insurance companies and the online food delivery companies to conduct surveillance of injuries and accurately evaluate the impact of occupational safety improvement programs for commercial delivery cyclists [21]. NSW road crash data indicates that 50 commercial delivery cyclists were injured in 2020; however, our study was able to identify at least 43 people injured at a single hospital over a 12-month period, suggesting that current reporting mechanisms might be underestimating the number of commercial delivery cycling injuries. Policy responses to a series of commercial delivery cyclist deaths in 2020 focussed on partnering with the industry to redesign the way work is performed, improve rider competency and equipment compliance, promote positive interactions between commercial delivery cyclists and the general public and support suitable route direction selection [21]. To demonstrate whether these interventions adequately target the characteristics of commercial delivery cycling injuries (e.g. high proportion of collision injuries sustained during the evening), improvements in data collection are required that make use of hospital ED presentations in addition to other sources of data.

In addressing the secondary aim of this pilot study, our findings are concordant with previous claims that much of the commercial delivery cycling workforce is comprised of temporary migrants on students visas in Australia [22]. In New South Wales, Australia, employees are entitled to a form of insurance payment if they are injured at work (i.e. workers’ compensation). However, many online food delivery companies classify their delivery cyclists not as employees but independent contractors who are responsible for their own insurance and other work-related costs. The implications of which appear to shift the funding of health-related costs from injuries obtained during the conduct of work from the workers’ compensation scheme to other funding mechanisms, such as compulsory third-party insurance for those injured in a motor vehicle incident or hospitals bearing the costs of Medicare ineligible patients (i.e. non-compensable). While some food delivery companies have introduced limited insurance schemes for delivery cyclists, the current study findings provide some support for the claim that treatment costs of injuries sustained while engaged in commercial cycling were likely to be subsumed by government, the injured individual or their private insurer, rather than the food delivery companies.

The higher rate of motor vehicle collisions experienced by commercial delivery cyclists in this study might indicate that different approaches to safety improvements are required for those cycling commercially compared to those who cycle recreationally. For example, emotions and personalities have long been studied in relation to motor vehicle driver safety [23], yet have only recently gained scientific attention in relation to cyclists [24]. Those who have experienced collisions with motor vehicles are reportedly more anxious and irritable, engage in more frequent risk-taking behaviours and make a higher number of mistakes compared to those who have not been involved in collisions [25]. Furthermore, while commercial cyclists experience lower levels of anger than non-commercial cyclists, this does not necessarily translate to less aggressive cycling behaviours because these behaviours are potentially being used as a strategy for meeting work demands rather than expressing their emotion [24, 26]. How then could we expect commercial delivery cyclists to respond to education and training programs, if behaviours are driven by underlying working conditions rather than road safety knowledge and emotions while riding?

The high proportion of motor vehicle collisions experienced within this cohort and the broader recreational cycling population indicates a need for improvements in road safety for vulnerable road users. Improved active transport infrastructure and street design, including integrated, connected and convenient facilities with physical separation from motor vehicles has been recommended from extensive international research [27, 28]. Injury from active transport is less likely to occur at lower motor vehicle speeds [29, 30], lending support for lower speed limits and traffic calming road modifications. Beyond infrastructure, enforcement of traffic laws that consider the position of vulnerable road users and target distracted drivers and drink driving have been identified as important factors accounting for the difference in road safety for vulnerable road users in high-income countries [31, 32]. Encouraging smaller vehicles may also offer an improved safety dividend for vulnerable road users, as larger vehicle size has been associated with more severe injury and death in collisions with both cyclists and pedestrians [33]. Finally, traffic education for road users might also contribute to improved safety. At the time of this study, equipment safety checks and road safety training and education was not routinely provided by online food delivery companies to commercial delivery cyclists, despite many being overseas visitors and having a primary language other than English. However, no high-quality evidence is available indicating whether equipment safety checks or training and education might reduce commercial delivery cyclists’ collision-related injuries, particularly as our findings indicate comparatively high levels of helmet use (mandated by law in Australia) and their cycling behaviour is thought to be incentivised by competition around speed of delivery [12].

This study was subject to several limitations. Medical records were limited to a single site over a 12-month period. Reliance on retrospective data from a single source (i.e. hospital data only) prevented the identification of injuries that may have occurred without a hospital visit. Future research could consider identifying injured cyclists from multiple sources (e.g. road crash statistics, work health and safety incident data, hospital emergency presentations and injury reports from online food delivery companies). The sample size and inability to categorise a substantial portion of cyclists as either commercial or non-commercial mean that the study findings should be interpreted with caution. Limited documentation of commercial cycling status constrained the ability to categorise a large proportion of the cycling injuries as either commercial or non-commercial. In response, multiple variables were treated conservatively and recorded as unknown where explicit detail was not available from the medical record. The injury characteristics were presented as high-level categorisations, such as the nature and body region of the injury, which potentially constrained the ability to determine differences in injury characteristics between commercial and non-commercial cyclists. Discerning a validated ISS was also limited to the participants drawn from the local trauma registry for whom the ISS had already been calculated (~33% of the included records). There were also other demographic-related variables that were not consistently available in the medical records, such as ethnicity and employment status that may have expanded the generalisability of our findings to other settings. Further research is required to understand the health-related impact of commercial cycling injuries more fully in Australia and internationally. A prospective observational study of injured cyclists across multiple sites is required to classify commercial and non-commercial injuries accurately and completely, which would be a pre-requisite to designing and evaluating programs to improve occupational safety for these workers.

## Conclusion

The growth of commercial cycling, particularly through online food delivery services, has raised concern regarding commercial cyclist safety. This pilot study highlighted the difficulties categorising cycling injuries presenting to hospital emergency departments as either commercial or non-commercial, using routinely collected administrative data and medical record review. Yet, key differences in the demographic, incident and injury characteristics between commercial and non-commercial cyclists were still able to be identified, which may have implications for efforts to improve road safety for these uniquely vulnerable road users. Key data gaps in the injury surveillance of commercial delivery cyclists can constrain the ability to accurately design and evaluate the impact of occupational safety improvement programs for this population. Therefore, hospital emergency department presentation data should be considered as an additional source of information to enhance existing injury surveillance efforts using road crash and work health and safety data. Improvements in the recording of cycling injury commercial status in patient medical records and hospital administrative data could enable future large-scale cohort studies to establish the extent and risk factors associated with commercial cycling.

## Data Availability

The datasets analysed during the current study are not publicly available due to ethical approval conditions that are designed to protect the potential re-identification of patients. Please contact the corresponding author regarding reasonable requests for data.

## Abbreviations

AOR: Adjusted odds ratio
CI: Confidence interval
US: United Sates
ED: Emergency department
AIS: Abbreviated Injury Scale
ISS: Injury Severity Score
GCS: Glasgow Coma Scale
SD: Standard deviation

## References

[1] Buehler R, Pucher J (2021). The growing gap in pedestrian and cyclist fatality rates between the United States and the United Kingdom, Germany, Denmark, and the Netherlands, 1990–2018. Transp Rev.

[2] Beck B, Cameron PA, Fitzgerald MC, Judson RT, Teague W, Lyons RA (2017). Road safety: serious injuries remain a major unsolved problem. Med J Aust.

[3] Kreisfeld R, Harrison JE (2019). Pedal cyclist deaths and hospitalisations, 1999-00 to 2015-16.

[4] Li C, Mirosa M, Bremer P (2020). Review of online food delivery platforms and their impacts on sustainability. Sustainability.

[5] Lee DJ, Ho H, Banks M, Giampieri M, Chen X, Le D. Delivering (in) justice: food delivery cyclists in New York city. In: Bicycle justice and urban transformation. London: Routledge; 2016. p. 114–29.

[6] Bernmark E, Wiktorin C, Svartengren M, Lewné M, Aberg S (2006). Bicycle messengers: energy expenditure and exposure to air pollution. Ergonomics.

[7] Dennerlein JT, Meeker JD (2002). Occupational injuries among Boston bicycle messengers. Am J Ind Med.

[8] Kulanthayan S, See LG, Kaviyarasu Y, Nor Afiah MZ (2012). Prevalence and determinants of non-standard motorcycle safety helmets amongst food delivery workers in Selangor and Kuala Lumpur. Injury.

[9] Goods C, Veen A, Barratt T (2019). “Is your gig any good?” analysing job quality in the Australian platform-based food-delivery sector. J Ind Relat.

[10] Maimaiti M, Zhao X, Jia M, Ru Y, Zhu S (2018). How we eat determines what we become: opportunities and challenges brought by food delivery industry in a changing world in China. Eur J Clin Nutr.

[11] Heyer JH, Sethi M, Wall SP, Ayoung-Chee P, Slaughter D, Jacko S (2015). Drawing the curtain back on injured commercial bicyclists. Am J Public Health.

[12] Wang Z, Jiang G, Neitzel R, Zheng W, Wang D, Xue X. Road safety situation of courier and take-out food delivery electric bike riders: a cross-sectional study in one municipality in China. 2020, PREPRINT (Version 1) available at Research Square 10.21203/rs.3.rs-31421/v1. 10.1080/15389588.2021.1895129 34432567

[13] Papakostopoulos V, Nathanael D (2021). The complex interrelationship of work-related factors underlying risky driving behavior of food delivery riders in Athens, Greece. Saf Health Work.

[14] da Silva DW, Andrade SM, Soares DFPP, Mathias TAF, Matsuo T, de Souza RKT (2012). Factors associated with road accidents among brazilian motorcycle couriers. Sci World J.

[15] Shin DS, Byun JH, Jeong BY (2019). Crashes and traffic signal violations caused by commercial motorcycle couriers. Saf Health Work.

[16] Chung Y, Song T-J, Yoon B-J (2014). Injury severity in delivery-motorcycle to vehicle crashes in the Seoul metropolitan area. Accident Anal Prev.

[17] The Abbreviated Injury Scale 2005 - Update 2008. Edited by: Gennarelli TA, Wodzin E. Barrington: Association for the Advancement of Automotive Medicine; 2008.

[18] Baker SP, O'neill B. (1976). The injury severity score: an update. J Trauma Acute Care Surg.

[19] Sternbach GL (2000). The Glasgow coma scale. J Emerg Med.

[20] Fitzgerald MC, Curtis K, Cameron PA, Ford JE, Howard TS, Crozier JA (2019). The Australian trauma registry. ANZ J Surg.

[21] Food Delivery Riders Safety Taskforce led by SafeWork NSW and Transport for NSW (2021). Working together to improve Food Delivery Rider safety: industry action plan 2021-2022.

[22] Convery E, Morse A, Fung B, Wodak S, Powell Z, Quinn V, et al. Work health and safety of food delivery workers in the gig economy. Sydney: Safework NSW; 2020.

[23] Zhang T, Chan AHS (2016). The association between driving anger and driving outcomes: a meta-analysis of evidence from the past twenty years. Accident Anal Prev.

[24] Oehl M, Brandenburg S, Huemer AK (2019). German bike messengers’ experiences and expressions of cycling anger. Traffic Inj Prev.

[25] von Below A (2016). Verkehrssicherheit von Radfahrern: analyse sicherheitsrelevanter Motive, Einstellungen und Verhaltensweisen.

[26] Lajunen T, Parker D (2001). Are aggressive people aggressive drivers? A study of the relationship between self-reported general aggressiveness, driver anger and aggressive driving. Accident Anal Prev.

[27] Lusk AC, Furth PG, Morency P, Miranda-Moreno LF, Willett WC, Dennerlein JT (2011). Risk of injury for bicycling on cycle tracks versus in the street. Inj Prev.

[28] Teschke K, Harris MA, Reynolds CC, Winters M, Babul S, Chipman M (2012). Route infrastructure and the risk of injuries to bicyclists: a case-crossover study. Am J Public Health.

[29] Tefft BC (2013). Impact speed and a pedestrian’s risk of severe injury or death. Accid Anal Prev.

[30] Helak K, Jehle D, McNabb D, Battisti A, Sanford S, Lark MC (2017). Factors influencing injury severity of bicyclists involved in crashes with motor vehicles: bike lanes, alcohol, lighting, speed, and helmet use. South Med J.

[31] Wegman F, Weijermars W, editors. Ten years sustainable safety in the Netherlands; an assessment. TRB 90th Annual Meeting, Washington DC: Transportation Research Board (TRB); 2011.

[32] Stimpson JP, Wilson FA, Muelleman RL (2013). Fatalities of pedestrians, bicycle riders, and motorists due to distracted driving motor vehicle crashes in the US, 2005–2010. Public Health Rep.

[33] Oikawa S, Matsui Y, Nakadate H, Aomura S (2019). Factors in fatal injuries to cyclists impacted by five types of vehicles. Int J Automot Technol.

